# Melatonin Upregulates Sodium Channel Nav1.5 in Cultured Neonatal Rat Cardiomyocytes

**DOI:** 10.33549/physiolres.935533

**Published:** 2025-12-01

**Authors:** Aleksandra DURKINA, Mikhail GONOTKOV, Arseniy FURMAN, Olesya BERNIKOVA, Ksenia SEDOVA, Valeria MIKHAILOVA, Ilya VELEGZHANINOV, Jan AZAROV

**Affiliations:** 1 Department of Cardiac Physiology, Institute of Physiology, Komi Science Center, Ural Branch, Russian Academy of Sciences, Syktyvkar, Russia; 2 Department of Biomedical Technology, Faculty of Biomedical Engineering, Czech Technical University in Prague, Kladno, Czech Republic; 3 Almazov National Medical Research Centre, Saint Petersburg, Russia; 4 Institute of Biology, Komi Scientific Centre, Ural Branch, Russian Academy of Sciences, Syktyvkar, Russia

**Keywords:** Cultured neonatal rat cardiomyocyte, Melatonin, Sodium channel, Sodium current

## Abstract

It has been previously demonstrated that melatonin exerts antiarrhythmic effects under conditions of ischemia and reperfusion *in vivo* by maintaining a relatively high conduction velocity in the myocardium. However, mechanistical details of this effect remain unclear, specifically whether melatonin affects myocardium directly or via systemic mediating signaling. The aim of the present study was to assess the impact of melatonin on the expression of genes encoding proteins potentially responsible for maintaining myocardial conduction in cultured cardiomyocytes. Ventricular cardiomyocytes isolated from neonatal rats were incubated with melatonin (100 μM) for 24 hours. Melatonin at a concentration of 100 μM enhanced the mRNA expression level of Scn5a gene and increased the amplitude of INa sodium current in cultured neonatal rat cardiomyocytes, but did not affect the parameters of steady-state activation and inactivation of INa. Thus, the present study demonstrated the direct effect of melatonin on sodium current in cardiomyocytes.

## Introduction

Ventricular tachycardia and/or fibrillation are life-threatening events, which could arise in congenital disorders or complicate cardiovascular diseases and syndromes, such as myocardial ischemia and heart failure. Antiarrhythmic prevention is very important, but notorious proarrhythmic effects of classical antiarrhythmic drugs limit their use [1], which necessitates development of new effective and safe preventive strategies. The mechanism of the fatal arrhythmias is based to a great extent on the impairment of myocardial conduction, which in turn depends significantly on sodium channel functioning. Consequently, the search for a novel signaling agent that targets specific ion channels related to electrical conduction presents an important research problem.

It is reported that G protein-coupled receptors can regulate the function of voltage-gated ion channels including sodium channel [2–4]. Among other signaling molecules, melatonin confers pleiotropic cardioprotective actions [5,6]. Advantageous effects of melatonin include suppression of arrhythmogenesis. Although melatonin modifies several electrophysiological targets, an enhancement of myocardial conduction appeared crucial for the antiarrhythmic action [7–9]. In the *in vivo* experiments, melatonin has been shown to enhance INa sodium current in the adult rat ventricular cardiomyocytes which accounted for the increasing conduction velocity [10]. However, important question remains unsolved, namely whether melatonin acts on cardiomyocytes directly or its effect is mediated by systemic mechanisms. Melatonin activates G protein-coupled receptors (MT1 and/or MT2), which are present in the myocardium [11]. It means that the direct effect of melatonin on cardiomyocytes is probable but has not been still confirmed. Understanding this mechanism is essential for appreciating the cardioprotective potential of melatonin and its potential therapeutic applications. Cultured neonatal rat cardiomyocytes provide a simplified, controlled model to study the direct cellular effects of melatonin on sodium channel activity. Thus, to address the question stated above, we studied the influence of melatonin on sodium channels in cultured neonatal rat cardiomyocytes.

## Materials and Methods

**Fig. 1:**
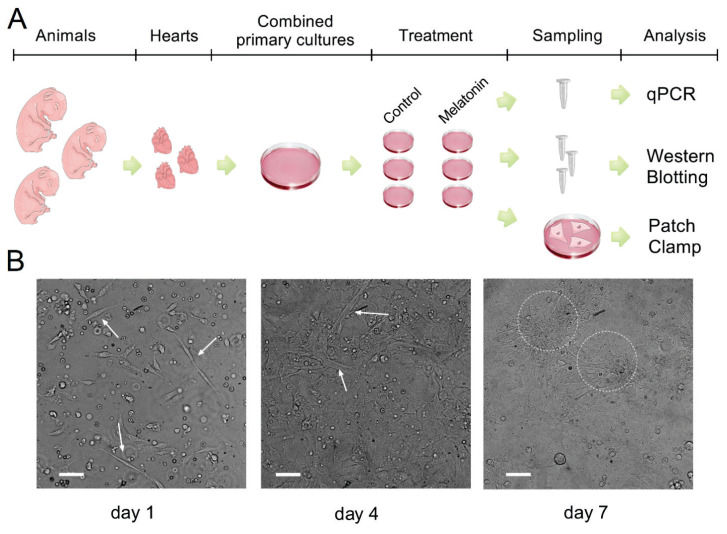
(**A**) Schematic for primary cultures of cardiomyocytes preparation and in vitro treatment with 100 μM melatonin to assess changes in the mRNA, protein levels of sodium channels and INa current. 2–3 protein samples were harvested from each treated culture (to increase the technical repetition of western blot analysis). One total RNA sample was isolated per culture for mRNA analysis using qPCR. For INa current recording using Patch-clamp each well of plate contained 2 to 3 coverslips with cells. (**B**) Contracting cells are indicated by arrows and dotted line. Scale bar 50 μm. 4 day neonatal CMs_2; 7 day neonatal CMs_2

In the present study, the hearts of newborn Wistar rats (1–3 days old) were used to obtain a primary cell culture of cardiomyocytes (CMs). The ventricles of 35 animals were isolated for the preparation of 9 combined cultures of cardiomyocytes. Each of the combined cultures was passaged in 2–3 small cultures (2–3 wells of 6-well plate). After 6–7 days of primary cells isolation cell, cultures were incubated with medium containing 100 μM melatonin (Sigma-Aldrich, USA) for 24 hours. Then RNA and total protein were extracted from the cells for studies using the RT-PCR method and for the western blotting analysis. The INa sodium current of cultured CMs was recorded using a whole cell patch-clamp technique. The study design is presented in Fig. 1A.

The study conformed to the Guide for the Care and Use of Laboratory Animals, Eighth Edition published by the National Academies Press (United States), 2011, the guidelines from Directive 2010/63/EU of the European Parliament on the protection of animals used for scientific purposes, and was approved by the ethical committee of the Institute of Physiology of the Komi Science Centre, Ural Branch of Russian Academy of Sciences (approval April 10, 2024).

### Isolation and cultures of neonatal ventricular cell

The protocol for isolation and culturing of cardiomyocytes was developed on the basis of several previously published ones [12–14]. Newborn rats were quickly decapitated (4–15 puppies per isolation). The hearts were collected in sterile calcium-free modified Krebs-Ringer’s solution on ice containing (in mM): 45 NaCl, 2.5 KCl, 1.25 NaH_2_PO_4_, 10 HEPES, 25 Glucose, 1 MgCl_2_, pH 7.4. All subsequent steps were performed under sterile conditions in a laminar-flow box. All solutions were prepared and sterilized by 0.2 μm filtration in advance. The hearts were transferred to a new tube with 200 μL of cold Krebs-Ringer’s solution, the atria and great vessels were carefully removed, and ventricles were homogenized using scissors. Then, 1 mL collagenase solution (Krebs-Ringer’s solution, 5 mg/mL collagenase Worthington, USA and 50 μM CaCl_2_) was added to the ventricular tissue, which was incubated for 9 min at 37 °C, 5 % CO_2_. The cell suspension was pipetted 30–40 times, and the supernatant was transferred to a new 2 mL tube. Cells were centrifuged at 1500 rpm for 10 minutes. The first supernatant fraction containing many fibroblasts and blood cells was discarded. After centrifugation, the cell pellet was resuspended in 1 mL of Dulbecco’s modified eagle’s medium (low glucose) (Servicebio, China) supplemented with 10 % FBS (Hyclone, USA), 10 μM sodium lactate (Sigma-Aldrich, USA) and 1 % penicillin-streptomycin (Servicebio, China). The cell suspension was pre-plated on 6-well plates for 1 hour at 37 °C, 5 % CO_2_ to remove fibroblasts. Then the cell suspension was collected into a common tube, centrifugated at 800 rpm for 10 minutes. The supernatant was removed, and the cell pellet was resuspended in fresh medium. The number of cells were determined, and 7*10^5 cells were plated per well of the plate for PCR and Western blotting analysis, while 2*10^5 cells were plated on coverslips for Patch-clamp recordings. After 24 hours, the media was changed to contain 3 % FBS to remove dead cells; thereafter the medium was changed every 2 days. In day 1 and day 4 cultures, single contracting cells and small contracting cell clusters were visible, respectively (Fig. 1B). On days 6 and/or 7, when the myocytes formed large clusters, and synchronized contraction of the entire culture was observed, the cells were ready for use.

### PCR analysis

Total RNA was extracted from the cells using Aurum Total RNA lysis solution (Bio-Rad, USA) and diaGene RNA extraction kit (Dia-M, Russia), following the manufacturer’s instructions. The integrity of extracted RNA was verified by agarose gel electrophoresis.

Quantification was performed using Qubit RNA BR Assay Kit and a Qubit fluorometer (Thermo Fisher Scientific, USA). First-strand cDNA was synthesized from total RNA using a reverse transcription with the MMLV RT kit (Evrogene, Russia), according to the manufacturer’s recommendations. The RT-PCR reactions were carried out using qPCRmix-HS SYBR (Evrogen, Russia) on QuantStudio 5 system (Thermo Fisher Scientific, USA). The following PCR cycling conditions were used: initial denaturation at 95 °C for 5 minutes, followed by 40 cycles of 95 °C for 15 seconds, 57 °C for 15 seconds, and 72 °C 30 seconds. Each analysis was performed in triplicate technical replicates, and the mean relative expression values from these replicates were used for subsequent statistical analysis. Relative expression was calculated using the ΔΔCt method [15] by normalizing to the geometric averaging of GAPDH gene and β-actin gene. Primers were designed using Primer-BLAST online tools. The sequences of the primers used for RT-PCR are as follows: Scn5a forward, 5′-TGTGTGCGT AACTTCACCGA-3′, reverse, 5′-ACATCCGTGGTGCC ATTCTT-3′; Scn1b forward, 5′-GTCACGTCTACCG TCTCCTC-3′, reverse, ′5′-GGCAGCAGCAATCT TCTTGT-3′; GAPDH forward, 5′-ATGGTGAAGGT CGGTGTGAA-3′, reverse, 5′-CGACATACTCAG CACCAGCAT-3′; ′β-actin forward, 5′-GCCTTCCTTCCT GGGTATGG-3′, reverse, 5′-ACGCAGCTCAGTA ACAGTCC-3′. Oligonucleotides were synthesized by Evrogen (Russia). The specificity of the primers was confirmed by agarose gel electrophoresis.

### Western blotting

The neonatal rat cardiomyocytes were washed with cold-ice PBS and lysed in 350 μL of RIPA buffer (Servicebio, China) and protease inhibitors cocktail (TransGen, China) to isolate total proteins. Lysate concentrations, purified by centrifugation, were measured using the Quick Start Bradford Protein Assay Kit (Bio-Rad, USA) on a CLARIOstar Plus plate reader (BMG Labtech, USA). For each sample, 40 mg of protein was separated on 8 % acrylamide gels (Smart-Lifesciences, China) and transferred to PVDF membranes (Bio-Rad, USA). Following blocking with 3 % bovine serum albumin (BSA) in TBST solution (0.1 % Tween-20, 150 mM NaCl, 20 mM Tris) for 1 hour, the membrane fragments were incubated at room temperature for 3 hours with primary antibodies Anti-NaV1.5 (Scn5a) Antibody (diluted 1:2000 in TBST with 3 % BSA, #PA5-115620, Thermo Fisher Scientific, USA) and Anti-Tubulin beta Antibody (diluted 1:2000 in TBST with 3 % BSA, #AF7011, Affinity Biosciences, China). After washing in TBST, the membranes were incubated for 1 hour at room temperature with recombinant Goat Anti-Rabbit IgG (H+L) HRP antibody (diluted 1:25000, #S0001, Affinity Biosciences, China). Subsequent to five washes in TBST, the Immun-Star Western C reagent and Chemidoc XRS imager (both from Bio-Rad, USA) were employed for blots visualization. Signal quantification from the recorded images was performed using the ImageLab software (Bio-Rad, USA). The Western blot analyses were conducted at the Centre of Collective Usage «Molecular biology» of the Institute of Biology of Komi SC UrB RAS.

### Patch-clamp electrophysiological recording

Whole-cell patch-clamp recordings of the sodium current (INa) were conducted using the Axopatch 200B amplifier (Axon Instrument, USA) under controlled room temperature conditions (22–24 °C). Coverslips with primary culture of neonatal rat cardiomyocytes were placed into the bath chamber perfused with a Cs+-based low-Na+ extracellular solution containing (in mM): 80 NaCl, 10 CsCl, 1 MgCl_2_, 2 CaCl_2_, 20 TEA-Cl, 20 glucose, 1.2 KH_2_PO_4_, and 10 HEPES, with the pH 7.4 adjusted using NaOH at a temperature of 22 °C. Patch pipettes with an average resistance of 1.6 ± 0.3 MΩ were pulled using a HEKA PIP 6 pipette puller (HEKA Electronics, USA) from borosilicate glass (Sutter Instruments, USA). These pipettes were filled with an intracellular solution containing (in mM): 10 NaCl, 130 CsCl, 1 MgCl_2_, 5 EGTA, 4 Mg_2_ATP, and 10 HEPES, with pH adjusted to 7.2 with CsOH. Prior to recording, pipette capacitance, cell capacitance, and series resistance were compensated to ensure accurate measurements. The INa current was recorded in the presence of 2 × 10–5 M nifedipine in the bath solution to inhibit ICaL. The INa current was obtained using a square-step protocol (−120 mV − +60 mV) from a holding potential of −120 mV with 10 mV steps applied every 2000 ms. The INa sodium current was recorded using software WinWCP 5.4.1 (Strathclyde Electrophysiology Software, UK), data processing was carried out using ClampFit 10.6 software (Molecular devices, USA). The steady-state voltage dependence of activation and inactivation for the voltage-gated sodium channels was determined by the Boltzmann equation, as described in [16].

### Statistical analysis

Statistical analyses were performed using GraphPad Prism 8.0 (GraphPad Software, USA). To test normality of data distribution, the Shapiro–Wilk test was used. Differences between groups were evaluated using unpair Student t-test. All data are presented as mean ± SEM, and the differences were considered significant at p<0.05.

## Results

### Expression of Scn5a mRNA and NaV1.5 protein in neonatal rat cardiomyocytes after melatonin treatment

**Fig. 2:**
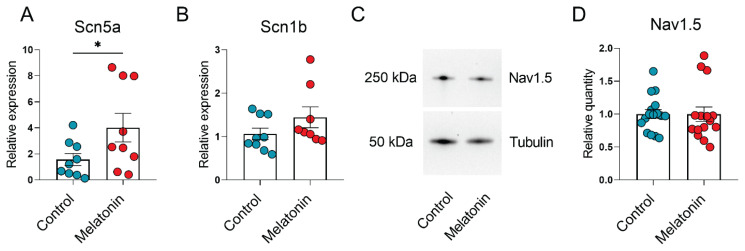
Expression of Na_V_1.5 sodium channel in cultured neonatal rat cardiomyocytes exposed to 100 μM melatonin. A, B - Relative mRNA expression of *Scn5a* (**A**) and *Scn1b* transcripts (**B**) in cultured neonatal rat cardiomyocytes, * p < 0.01. C – Representative blots: exposure time for filming of Nav 1.5 was 1137 sec, for Tubulin – 56 sec. (**C**) and relative quantity of Nav1.5 protein (**D**) encoded by Scn5a. The data are presented as mean ± SEM, unpaired Student t tests.

The relative mRNA expression of Scn5a gene transcript was significantly increased in cardiomyocytes after incubation with melatonin at a concentration of 100 μM (Fig. 2A) compared to the control group. The relative mRNA expression of Scn1b gene transcript did not differ between the groups.

Interestingly, western blotting provided contrasting results regarding the expression of NaV1.5 protein in neonatal cardiomyocytes at the same concentration of the melatonin when compared to the relative mRNA expression. Specifically, the level of NaV1.5 protein in cultured cells remained unchanged after 24-hour exposure to melatonin, similar to the control group (Fig. 2B).

### Melatonin increases the amplitude of INa current in cultured neonatal rat cardiomyocytes

**Fig. 3:**
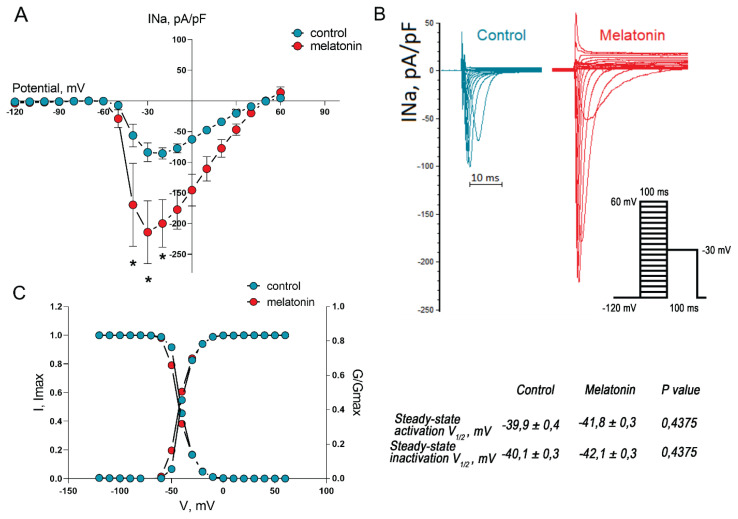
Effect of melatonin on INa current in cultured neonatal rat cardiomyocytes. (**A**) - The current-voltage (I–V) relationship in cardiomyocytes with and without melatonin supplementation (100 μM, 24 hours) demonstrates that melatonin increases INa, * p<0.01. (**B**) - Representative traces of INa in the control and melatonin groups (voltage protocol shown in the inset). (**C**) – The steady-state activation and inactivation curves of INa showed no statistically significant differences between the groups. The data are presented as mean ± SEM, unpaired Student t-tests.

Representative whole-cell traces and I–V curves illustrated the increase in the amplitude of INa in neonatal cardiomyocytes incubated with melatonin (Fig. 3A,B). Interestingly, this increase in amplitude was not caused by changes in their gating properties since steady-state activation and inactivation curves did not differ in the treated and control cells (Fig. 3C).

## Discussion

In the present study, we demonstrated that melatonin influences sodium channels in cultured neonatal cardiomyocytes. Specifically, 24-hour melatonin incubation enhanced the expression of the Scn5a gene and significantly increased the INa sodium current in rat neonatal cardiomyocytes. However, this treatment did not affect the characteristics of steady-state activation and inactivation of the sodium channels and the overall level of Nav1.5 protein.

Previous research has demonstrated that melatonin treatment *in vivo* reduces the occurrence of life-threatening ventricular tachyarrhythmias *in vivo* [7–9], shortens myocardial activation time, and enhances INa current as well as mRNA of Scn5a gene transcripts and Nav1.5 protein expression in the ventricles of adult rats [10]. The plausible explanation for the antiarrhythmic effect of melatonin is maintaining conduction velocity in the affected myocardium, which prevents reentry circuit development. The results of the present study are consistent with the above-mentioned *in vivo* findings concerning the expression of Scn5a gene mRNA and INa current. However, we did not observe the changes in the relative protein levels of Nav1.5, which might be accounted for by a suggestion that in addition to the increase in Scn5a expression melatonin caused posttranslational effects on Nav1.5 that were not reflected in the changes in the protein level but were expressed functionally as the increase in INa current. It also cannot be excluded that the expected effect on the relative protein levels of Nav1.5 appeared to be not high enough to be detected by the used technique and setting. This explanation is supported by our previous observations [10] of the increased Nav1.5 level after a longer (7 days) melatonin treatment *in vivo*.

The effects of melatonin on the sodium channels are expected to be mediated by complex melatonin signaling pathways. Since both MT1 and MT2 melatonin receptors are predominantly coupled to Gαi/o subunits, they typically signal through inhibition of the cAMP/PKA pathway [17] thereby suppressing transcription of genes controlled by the cAMP responsive element binding protein [18]. However, recent research revealed other possible signaling pathways for melatonin to influence the heart. Activation of melatonin receptors can either decrease or increase cAMP production depending on the relative abundance of Gs and Gi proteins, MT1 and MT2 receptors, and on the efficacy of the respective receptor/G proteins coupling [19]. Melatonin can directly and indirectly interact with calmodulin and affect calmodulin distribution in the cellular compartments [20–22]. These interactions account for both inhibition (mostly short-term) and activation (mostly long-term) of the CaM-dependent pathways by melatonin. Also, melatonin activates PI3K/Akt and ERK1/2 pathways via MT1 and/or MT2 receptors [23,24] and therefore positively regulate transcription of genes controlled by these pathways [18].

Many of the melatonin signaling pathways delineated above can be involved in the regulation of Nav1.5 channel expression and function. Sodium channels can be regulated by multiple kinases, in particular protein kinase A, protein kinase C and calcium/calmodulin-dependent kinase II, which phosphorylate and regulate the biosynthesis of Nav1.5 channels [25]. Most of these regulation pathways cause not only the change in the INa amplitude, but also modification of channel gating. The latter was unaffected by melatonin in our study. Phosphorylation of Ser671 residue in the intracellular loop of Nav1.5 can change the protein expression in the plasma membrane and the current peak density without modification of channel gating characteristics [26]. However, it is unclear whether this phosphorylation site could be affected by the melatonin-dependent signaling pathways. On the other hand, since Scn5a expression can be enhanced by the activation of Akt/ERK1/2-FOXO1 signaling pathway [27] and melatonin activates it [18], the observed increase in Scn5a expression and INa current might be due to Akt/ERK1/2 involvement. Taken together, the data imply that melatonin could affect sodium channels on both expression and posttranslational levels via different signaling pathways and these influences could account for our findings. However, direct evidence on details of these pathways is still lacking and should be further elucidated.

The changes in sodium current might have been also caused by the involvement of other sodium channel isoforms. However, most of these isoforms are not expressed at a significant level in ventricular cardiomyocytes. Although Nav1.1 is present in the myocardium, it primarily contributes to the late rather than fast sodium current [28]. In our study, no changes were observed in the steady-state activation and inactivation characteristics of sodium channels, suggesting that the increase in peak INa is mediated by Nav1.5 rather than by the other isoforms. It is also noteworthy that in the present study melatonin did not affect expression of Scn1b encoding the β-subunit of the sodium channel responsible for the regulation of the kinetics and gating of the sodium channels [29, 30]. It is consistent with the unchanged steady-state activation and inactivation characteristics of the sodium channel in our experiments.

### Study limitations

Neonatal rat ventricular myocytes serve as a good experimental model. However, despite multiple advantages, several disadvantages are present. Neonatal cardiomyocytes have an immature cellular phenotype, which can manifest in strong variability of electrophysiological parameters and data scatter (we observed variations in cultures consisting of cells from several hearts). Nevertheless, all primary cultures in our study were obtained according to a uniform well-established protocol and we were able to demonstrate consistent melatonin effects at least concerning Scn5a expression and characteristics of INa current. Finally, we did not conduct direct measurements of intracellular kinases or other secondary messengers to fully determine the precise mechanisms of melatonin action for melatonin. Therefore, the discussed mechanisms remain speculative and need to be further investigated.

## Conclusion

In this study for the first time, we demonstrated the ability of melatonin to augment sodium current in cultured neonatal rat cardiomyocytes due to its direct action on cells independently of its possible systemic effects in the organism. Notably, melatonin did so without altering the sodium channel gating, which is important because alterations in steady-state activation and inactivation can lead to an increase in an arrhythmogenic late sodium current. Our data suggest that melatonin impact on cardiac electrophysiology is multifaceted, with direct effects on sodium currents that could have implications for cardiac impulse conduction.

## Supplementary Information
